# Evolution of *TP53* abnormalities during CLL disease course is associated with telomere length changes

**DOI:** 10.1186/s12885-022-09221-z

**Published:** 2022-02-03

**Authors:** Helena Olbertova, Karla Plevova, Sarka Pavlova, Jitka Malcikova, Jana Kotaskova, Kamila Stranska, Michaela Spunarova, Martin Trbusek, Veronika Navrkalova, Barbara Dvorackova, Nikola Tom, Karol Pal, Marie Jarosova, Yvona Brychtova, Anna Panovska, Michael Doubek, Sarka Pospisilova

**Affiliations:** 1 Central European Institute of Technology, Masaryk University, 625 00 Brno, Czech Republic; 2 Department of Internal Medicine Hematology and Oncology, University Hospital Brno and Faculty of Medicine, Masaryk University, Brno, Czech Republic; 3 Department of Medical Genetics and Genomics Faculty of Medicine, Masaryk University and University Hospital Brno, Brno, Czech Republic

**Keywords:** Chronic Lymphocytic Leukemia, Telomere, *TP53*, Clonal evolution, BCR signaling

## Abstract

**Background:**

Telomeres are protective structures at chromosome ends which shorten gradually with increasing age. In chronic lymphocytic leukemia (CLL), short telomeres have been associated with unfavorable disease outcome, but the link between clonal evolution and telomere shortening remains unresolved.

**Methods:**

We investigated relative telomere length (RTL) in a well-characterized cohort of 198 CLL patients by qPCR and focused in detail on a subgroup 26 patients who underwent clonal evolution of *TP53* mutations (evol*TP53*). In the evol*TP53* subgroup we explored factors influencing clonal evolution and corresponding changes in telomere length through measurements of telomerase expression, lymphocyte doubling time, and BCR signaling activity.

**Results:**

At baseline, RTL of the evol*TP53* patients was scattered across the entire RTL spectrum observed in our CLL cohort. RTL changed in the follow-up samples of 16/26 (62%) evol*TP53* cases, inclining to reach intermediate RTL values, i.e., longer telomeres shortened compared to baseline while shorter ones prolonged. For the first time we show that *TP53* clonal shifts are linked to RTL change, including unexpected RTL prolongation. We further investigated parameters associated with RTL changes. Unstable telomeres were significantly more frequent among younger patients (*P* = 0.032). Shorter telomeres were associated with decreased activity of the B-cell receptor signaling components p-ERK1/2, p-ZAP-70/SYK, and p-NFκB (*P* = 0.04, *P* = 0.01, and *P* = 0.02, respectively).

**Conclusions:**

Our study revealed that changes of telomere length reflect evolution in leukemic subclone proportion, and are associated with specific clinico-biological features of the explored cohort.

**Supplementary Information:**

The online version contains supplementary material available at 10.1186/s12885-022-09221-z.

## Background

Chronic lymphocytic leukemia (CLL) is a biologically heterogeneous entity with variable clinical outcomes [1–3]. CLL tumor cell mass can be generally composed of multiple genomically distinct subclones endowed with different capacities to proliferate and survive, and their relative composition evolves during the disease course, especially under therapy pressure [4, 5]. This process, generally known as clonal evolution, crucially drives CLL disease progression. Nowadays, many established prognostic biomarkers are used to stratify CLL patients into clinically distinct subgroups. Among these, the aberrations affecting the *TP53* gene have a particularly adverse effect on patient prognosis and chemotherapy response [6, 7]. *TP53* defects frequently arise in CLL relapse after chemoimmunotherapy despite being undetectable at the time of diagnosis [8, 9] and are strongly associated with chemotherapy resistance [10, 11]. Therefore, the patients with aberrant *TP53* should be treated with novel therapeutics based on BCR signaling or bcl-2 inhibition [12–15].

Telomeres are complex repetitive DNA sequences bound by multiple interacting proteins, which protect ends of linear chromosomes against end-to-end chromosomal fusion and degradation. Human telomeres are composed of ∼4–11 kb long hexameric 5 ‘-TTAGGG-3 ‘ repeats in lymphocytes [16, 17], and their maintenance strategies are cell-type specific. While telomeres of somatic cells shorten with each cell division, germ cells are capable of maintaining stable telomere length using telomerase to sustain cell immortality [18]. However, even in stem cells, telomerase activity is limited and when eventually exhausted, it causes senescence and loss of ability to renew tissues [19, 20]. Of note, telomeres of normal B cells are naturally prolonged by telomerase during their activation in a germinal center of secondary lymphoid tissues [21].

Cancer cells are also able to activate telomerase to overcome short telomeres to ensure cell immortality [22]. In CLL, short telomeres have been associated with markers of inferior prognosis, such as unmutated IGHV genes, increased genomic complexity, 11q deletion/mutated *ATM* gene, and 17p deletion/mutated *TP53* gene [23–30], with short time to first treatment [31] and short overall survival (OS) [17, 27, 32, 33]. Telomere length is considered stable in most CLL cases; however, its changes have been observed in a subset of follow-up samples, especially in those undergoing clonal evolution [27, 34]. Furthermore, the level of telomerase activity in CLL cells is similar to its level in normal B lymphocytes, although a subgroup of IGHV-mutated CLL cells had a significantly lower telomerase activity than healthy B cells [23]. In some studies, high telomerase activity was observed and correlated with poor prognosis [31, 35].

This present study follows up our previous efforts focused on investigating clonal evolution of *TP53* aberrations [8] and on the associations of *ATM* aberrations with short telomere length [29] in CLL. Here we explored a well-characterized cohort of 198 CLL patients to assess associations between CLL genomic features and telomere length. Sequential samples from 26 patients, who underwent clonal evolution of *TP53* mutations (evol*TP53* group) over the disease course, revealed for the first time an interplay between telomere length of subclones, BCR signaling activity, and CLL clinico-biological features.

## Methods

### Primary CLL samples

Two hundred and thirty-five CLL samples from 198 CLL patients were included in the present study; clinical and laboratory data were available for all tested cases (Tables 1 and 2). CLL cells were separated from peripheral blood with high purity (> 98%) as we described previously [36] using Ficoll-Paque PLUS (GE Healthcare, USA) combined with RosetteSep Human B cell Enrichment Kit and CD3 + Depletion Kit (StemCell Technologies, Canada) to deplete non-B cells.

The study was approved by the Ethical Committee of the University Hospital Brno and all patients provided informed consent to the research use of biological material and clinical data in agreement with the Helsinki declaration.

### Molecular characterization of the cohort

IGHV gene somatic hypermutation status was analyzed as described previously [37, 38].

Exonic regions and splice sites of the *TP53* and *ATM* genes were amplified using in-house designed primers [8, 29]. Sequencing libraries were prepared using Illumina Nextera XT library preparation kit (Illumina, USA) and sequenced using Illumina MiSeq or NextSeq machines in paired-end mode (2 × 150 bp). The results were analyzed with an in-house bioinformatic pipeline [8, 29]. The cut-off variant allelic frequency (VAF) of 10% recommended in routine clinical diagnostics [39] was used to identify *TP53* and *ATM* mutated cases. However, for the characterization of the evol*TP53* group, ultra-deep sequencing with a cut-off 0.1% VAF was used to confirm *TP53* clonal evolution [8].

Genomic aberrations were assessed by FISH for del(11q), del(13q), del(17p), and trisomy 12 (XL CLL Probe Kit, MetaSystems). Genome-wide copy number and heterozygosity profiles were obtained using CytoScan HD arrays (Thermo Fisher Scientific). Metaphase cytogenetics was performed on peripheral blood samples (*n* = 169) stimulated with IL 2 and DSP30 CpG Oligonucleotide. Complex karyotype and highly complex karyotype were defined as ≥ 3 or ≥ 5 abnormalities, respectively.

### Relative telomere length (RTL) measurement

Telomere length was measured by the quantitative polymerase chain reaction (qPCR) method described previously [29, 40] with certain modifications (see Supplementary Material) using primers specific for telomeric repetition [41]. HBG3 and HBG4 primers [42] were used for the amplification of β-globin (HBB) gene, serving as a single-copy gene control.

Each sample was measured in triplicates for both RTL and HBB, the reactions were assembled by Freedom EVO automated system (Tecan, Switzerland) in 384-well plates, and qPCR was performed by QuantStudio 12 k Flex (Applied Biosystems, USA). Each reaction contained 15 ng DNA, 100 nM F and R primers, 5 mM DTT, 10 mM Tris–HCL (pH 8), 130 mM DMSO, 1.5 µM BSA, 50 nM ROX in the final volume 10 µl. Quantitative PCR was performed on QuantStudio 12 k Flex (Applied Biosystems) with the cycling conditions as follows: 10 min 95 C, 40x (20 s 95 °C, 1 min 56 C). The RTL was calculated as -ΔCT for CLL samples divided by -ΔCT for control human DNA sample (G3041, Promega, USA) isolated from leukocytes of healthy donors. The -**ΔCT** values represent the difference between average Ct values of telomere length and HBB gene. The cut-off value for RTL change in consecutive CLL cases was 5% difference of RTL between baseline and follow-up, which corresponded to the complete loss of telomeres on two chromosomes (calculated approximately as 46 chromosomes with 2 telomeric regions each = 92 telomeric regions, 5% loss corresponds to loss of ~ 4 telomeric regions).

### Human telomerase reverse transcriptase (hTERT) expression

Reverse transcription was carried out with SuperScript II Reverse Transcriptase (Thermo Fisher Scientific). One ng of reverse-transcribed cDNA was used for real-time PCR containing 2 × ABsolute QPCR Mix (low ROX, Thermo Fisher Scientific) and either hTERT or GAPDH TaqMan assay (Hs00972650_m1 for hTERT, Hs03929097_g1 for GAPDH, Thermo Fisher Scientific) following the manufacturer protocol. PCR was performed on QuantStudio 12 k Flex (Applied Biosystems) with the cycling conditions: 15 min 95 °C, 40x (15 s 95 °C, 1 min 60 °C).

### BCR signaling assessment

Fresh frozen CLL cells were thawed, fixed, and permeabilized with 1.6% paraformaldehyde and 100% methanol. Supplementary Table 1 shows that the samples were incubated with antibodies against phosphorylated AKT, BTK/ITK, ERK1/2, IKKα/β, NF-κB, p38, PLCγ2, and ZAP-70/SYK at 20 °C for 60 min, and measured using FACSVerse flow cytometer (BD Biosciences). Median fluorescent intensity (MFI) value, representing the amount of basal phosphorylation of BCR signaling pathway components, was used for further analysis.

### Statistical analysis

All statistical analyses were performed using software JASP (Version 0.11.1) [43]. Specific statistical tests used for different study variables are described in the figure legends. T-tests were Student’s two-tailed, RTLs of paired samples were evaluated by Wilcoxon signed rank test, correlations were counted as Pearson’s r. *P* values < 0.05 were considered statistically significant.

### Data sharing statement

For the original data and detailed protocols please contact the corresponding author.

## Results

### Telomere length associates with clinical features of the disease

In order to comprehensively assess the association between genomic features and telomere length in CLL, we studied a set of 198 clinically and genetically well-characterized CLL cases (Table 1). The majority of samples (*n* = 153; 77.3%) was collected prior to first treatment, and for patients with multiple available samples, only the first sample was included here. Forty samples (20.2%) carried a *TP53* mutation above 10% VAF (with concomitant del(17p) in 23/40 cases).

**Table 1:** Composition of the basic CLL cohort (*n* = 198)

Parameter	*n* (%)
Sex (female/male)	77/121 (38.9% / 61.1%)
Age at diagnosis (median)	62.4 years
Untreated at the time of analysis	153 (77.3%)
Time to first treatment (median; *n* = 182)	30 months
IGHV status (mutated/unmutated)	79/119 (39.9% / 60.1%)
Hierarchical cytogenetics (FISH)	
del(17p)	28 (14.1%)
del(11q)	45 (22.7%)
trisomy 12	18 (9.1%)
normal	37 (18.7%)
del(13q)	70 (35.4%)
Complex karyotype (≥ 3 changes; *n* = 169)	47 (27.8%)
Highly complex karyotype (≥ 5 changes; *n* = 169)	23 (13.6%)
* ATM* mutation > 10% VAF (*n* = 191)	37 (18.9%)
* TP53* mutation > 10% VAF	40 (20.2%)

The median RTL of the entire CLL cohort was 0.81 (range 0.46 – 1.25; Supplementary Figure 1), meaning that telomere length in CLL cells was generally shorter than in the control mixed human DNA sample. Additionally, we observed significantly longer RTLs in patients at Rai and Binet early disease stages at diagnosis (Supplementary Figure 2). To assess the impact of RTL on OS, we split the cohort into two groups with RTLs above and below median RTL (median RTL = 0.84) and detected statistically significant difference between the groups (median OS 119 and 91 months, respectively; *P* = 0.0004; Supplementary Figure 3A). When focusing the analysis on distinct patient subgroups with genomic aberrations, we found statistically significant associations of RTL with unmutated IGHV somatic hypermutation status, recurrent cytogenetic aberrations in hierarchical order [44], complex karyotype (as defined by 3 abnormalities), and mutations in *TP53* and *ATM* genes (Supplementary Table 2), which was in line with previously published results [23–26, 28, 33] thus confirming the representativeness of the patient cohort. Since 22.7% of samples were obtained after treatment, we tested whether RTL is dependent on treatment administration. In agreement with Mansouri et al. [34]*,* we observed that previous therapy was not significantly associated with RTL (Supplementary Table 2). Nonetheless, the treated and untreated cohorts were not fully biologically and clinically comparable.

### Clonal evolution of TP53 defects affects telomere length

We focused in detail on the associations between telomere length and *TP53* mutational status in our CLL cohort. First, we evaluated the impact of RTL on patient OS in the subgroup with *TP53* mutations (*n* = 40). We observed that OS of this subgroup divided by the RTL value above and below median was statistically different favoring cases with longer RTL (median RTL = 0.78; median OS 86 and 55 months, respectively; *P* = 0.046; Supplementary Figure 3B).

**Fig. 1:**
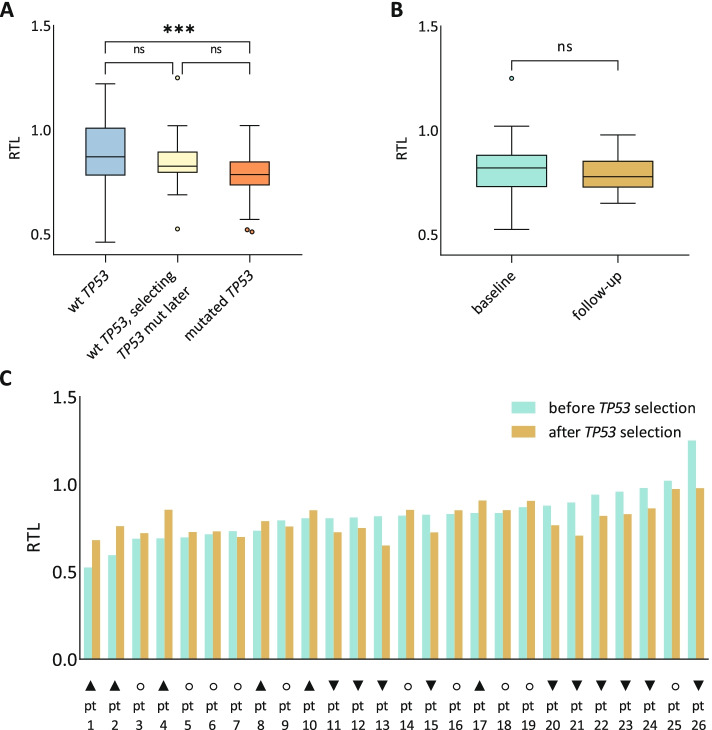
Relative telomere length dynamics in the context of *TP53* mutation clonal evolution. **A)** The analysis of the RTL and *TP53* mutation status (the cut-off VAF 10%) within the basic CLL cohort (*n* = 198), showing a difference between the subgroups of patients characterized by wt*TP53* (blue; *n* = 135), patients expanding mut*TP53* above 10% VAF later in a disease course (yellow; *n* = 23), and mut*TP53* (red; *n* = 40). **B)** RTLs in baseline and follow-up samples in the evol*TP53* cohort (*n* = 26; Wilcoxon signed rank *P* = 0.291) **C)** RTL change in individual patients in the evol*TP53* cohort. Telomeres became shorter in 10/26 (38.5%), remained stable in 10/26 (38.5%), or became longer in 6/26 (23.1%) patients. Higher RTL shrank and lower RTL extended with an inverse relationship during the disease course (Pearson r = -0.75, *P* < 0.001). RTL change marks: ▼shortening, ○ stable, ▲ prolonging. The cut-off value for RTL change was set to 5% difference of RTL between baseline and follow-up samples

Next, available retrospective ultra-deep NGS data [39] were used to divide the patients from the basic cohort into three groups: (I) patients with wild-type *TP53* (wt*TP53*; *n* = 135; mutation status has not changed throughout the observation), (II) patients with mutated *TP53* above 10% VAF (mut*TP53*; *n* = 40; median VAF = 57.5%), and (III) patients with *TP53* mutation expanding above 10% VAF later in a disease course (*n* = 23). All group III patients except one expanded a *TP53* mutation after therapy. Median *TP53* VAF of group III was 0.2% (0% – 5.9%) at baseline and 43.4% (12.1%—100%) at follow-up, corresponding to median fold change of 211. Figure 1 summarizes the RTL dynamics in the context of *TP53* mutation clonal evolution. When we compared RTLs between the patients with wt*TP53* (group I) and mut*TP53 (*group II), we found that the mut*TP53* samples had significantly shorter telomeres (median RTL 0.87 and 0.78, respectively; *P* < 0.001; Fig. 1A). Interestingly, RTLs of group III (with *TP53* mutation expanding above 10% VAF later) were intermediate (median RTL 0.83; Fig. 1A) and they did not differ significantly either from group I (*P* = 0.25) or group II (*P* = 0.10).

**Fig. 2:**
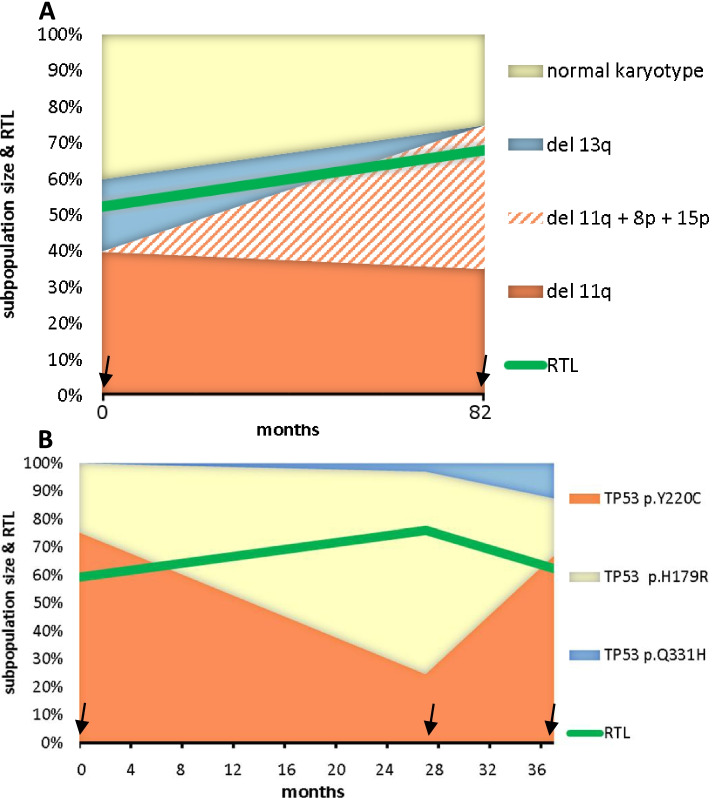
Examples of RTL change associated with shifts in a proportion of clones bearing different aberrations. **A)** In the patient pt1, the RTL increased in disease relapse (the follow-up sample collected 81 months after the baseline sample, arrows indicate each sampling). The proportion of a new clone bearing del(11q), del(8p), del(15p), and a *TP53* mutation increased to approximately 40% of the CLL population in the follow-up sample. At the same time, the proportion of the clone with sole del(11q) shrunk to approximately 35% of the CLL population and the clone with del(13q) disappeared. Concurrently with these changes of clonal proportions, higher RTL was detected. **B)** In the patient pt2 studied in three time points with three distinct clones carrying different *TP53* mutations, telomeres initially lengthened and subsequently shortened, closely following an exchange of a dominating *TP53* mutation (the first follow-up sample was collected 27 months after the baseline sample and the second follow-up sample was collected 10 months later, arrows indicate each sampling)

Apart from the above-mentioned 23 cases from group III manifesting *TP53* mutation expansion, we identified another 3 cases from group II (mut*TP53*) who experienced replacement of a certain *TP53* mutation by another one after therapy. Thus, 26 cases out of the entire cohort experienced *TP53* mutation evolution (evol*TP53* group) during the disease course (Table 2, Supplementary Table 3). In order to discover whether mut*TP53* evolution showed any association with telomere length, we studied pre- and post-evolution samples. All evol*TP53* cases were analyzed in at least 2 time points. The median follow-up between the pre- and post-evolution samplings was 38.5 months (range 18 – 122 months). The median RTL was 0.82 (range 0.52 – 1.25) before and 0.78 (range 0.65 – 0.98) after the mut*TP53* evolution (Fig. 1B). When focusing on individual patients, the RTL change course was inconsistent. In detail, we observed that telomeres became shorter, remained stable, or became longer in 10/26 (38.5%), 10/26 (38.5%), and 6/26 (23.1%) patients, respectively (Fig. 1C). Interestingly, short telomeres became longer over time, while in contrast, long telomeres became shorter (Pearson r = -0.75, *P* < 0.001; Fig. 1C). Additionally, for 6 and 2 of the 26 patients, 3 and 4 time points were analyzed, respectively, to follow further RTL development. Evolutions of genomic aberrations related to changes of RTLs in two CLL cases with the shortest basal telomere length are illustrated in Figs. 2A and 2B. Importantly, the apparent correlation between RTL and genomic abnormalities indicates that telomere length was likely a feature of CLL clones, not a hallmark of the entire CLL population changing in time.

**Table 2:** Composition of the CLL cohort evolving *TP53* mutation during the disease course, which underwent the telomere length measurements in multiple time points (*n* = 26)

Parameter	*n* (%)	
Sex (female / male)	12 / 14 (46.2% / 53.8%)	
Median age at diagnosis	57.7 years	
Median time to first treatment	20.0 months	
IGHV status (mutated /unmutated)	2 / 24 (7.7% / 92.3%)	
	baseline sample	follow-up sample
Previously treated for CLL	5 (19.2%)	26 (100%)
Hierarchical cytogenetics (FISH)		
del(17p)	3 (11.5%)	10 (38.5%)
del(11q)	10 (38.5%)	9 (34.6%)
trisomy 12	3 (11.5%)	2 (7.7%)
normal	6 (23.1%)	4 (15.4%)
del(13q)	4 (15.4%)	1 (3.8%)
Copy-neutral loss of heterozygosity 17p (*n* = 21)	0 (0%)	5 (23.8%)
Complex karyotype (≥ 3 changes; *n* = 24)	7 (29.2%)	9 (37.5%)
Highly complex karyotype (≥ 5 changes; *n* = 24)	4 (16.7%)	6 (25.0%)
* ATM* mutation > 10% VAF (*n* = 22)	4 (15.4%)	3 (12.5%)
* TP53* mutation > 10% VAF	3 (11.5%)	26 (100%)

### Association of RTL dynamics with clinico-biological features in cases evolving TP53 mutations

#### hTERT expression

Since the changes of telomere length could potentially be caused by deregulated telomerase activity [31, 45], we tested hTERT expression in the 26 evol*TP53* patients. However, only weak or null hTERT expression was detected. Supplementary Figure 4 and Supplementary Table 3 show that we did not find any correlation with telomere length or its change.

#### Lymphocyte doubling time (LDT)

Intensive cell division, which could possibly cause RTL shortening, can be estimated from LDT [24]. Supplementary Figure 5 and Supplementary Table 3 show the correlation of LTD of the evol*TP53* cases with the change of RTL between the baseline and follow-up. Short doubling time was observed in available baseline samples (median 6.5 months, range 0.5 – 39.6; *n* = 21) as well as in follow-up samples (median 2.6 months; range 0.4 – 81.5; *n* = 25). Nevertheless, no significant association of LDT and change of RTL was found.

#### BCR signaling activity

**Fig. 3:**
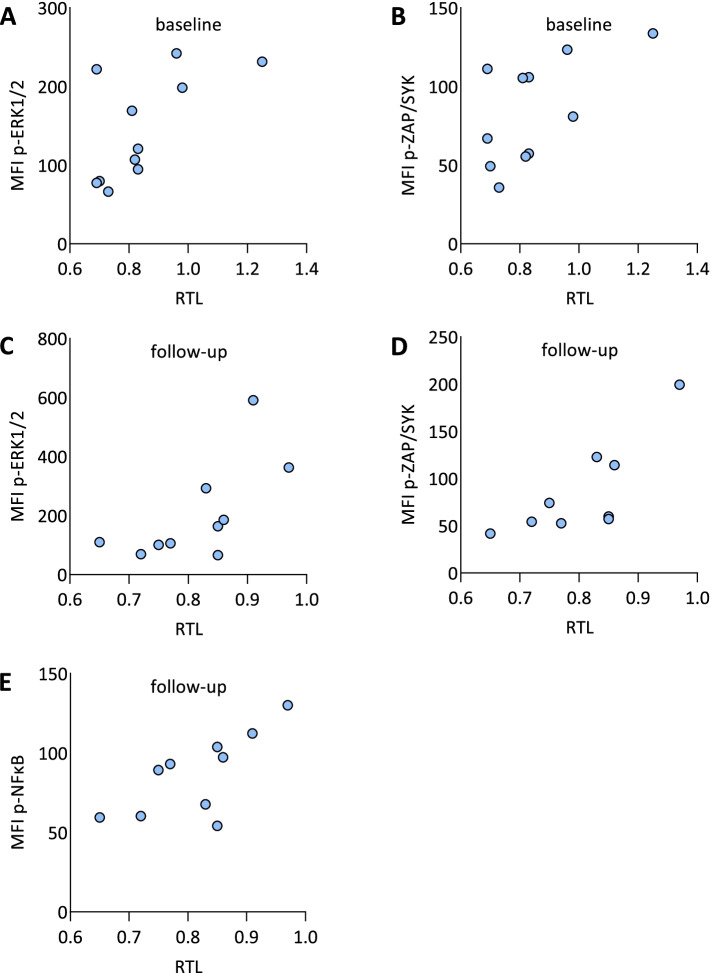
Relative telomere length dynamics and BCR signaling activity. Prior clonal evolution, RTL was **A)** lower in CLL samples with less phosphorylated ERK1/2 (Pearson r = 0.63; *P* = 0.04) and **B)** less phosphorylated ZAP-70/SYK (Pearson r = 0.60; *P* = 0.05). In later stages, RTL was **C)** lower in CLL cases with less phosphorylated ERK1/2 (Pearson r = 0.66; *P* = 0.04), **D)** less phosphorylated ZAP-70/SYK (Pearson r = 0.79; *P* = 0.01), and **E)** less phosphorylated NFkB (Pearson r = 0.70; *P* = 0.02)

To study the relation between RTL dynamics and the activity of BCR signaling, we applied flow cytometry to detect basal phosphorylation of selected intracellular markers in CLL cells obtained prior and after mut*TP53* expansion (*n* = 11 and 10, respectively, of these 7 samples were paired; Supplementary Table 3). Figure 3 illustrate the correlation of RTL and BCR signaling activity. Significantly longer telomeres were detected prior as well as after mut*TP53* expansion in cases with higher phosphorylation of ERK1/2 (prior mut*TP53* expansion: Pearson r = 0.63; *P* = 0.04; Fig. 3A; after mut*TP53* expansion: Pearson r = 0.66; *P* = 0.04; Fig. 3C; Supplementary Table 3) and ZAP-70/SYK (prior mut*TP53* expansion: Pearson r = 0.60; *P* = 0.05; Fig. 3B; after mut*TP53* expansion: Pearson r = 0.79; *P* = 0.01; Fig. 3D; Supplementary Table 3). Additionally, significantly longer telomeres after mut*TP53* expansion were detected in cases with higher phosphorylation of NF-κB (Pearson r = 0.70; *P* = 0.02; Fig. 3E; Supplementary Table 3). Moreover, the phosphorylation of ZAP-70/SYK and ERK1/2 closely correlated mutually (Pearson r = 0.82; *P* = 0.002 in baseline samples; Pearson r = 0.92; *P* < 0.001 in follow-up samples; Supplementary Figure. 6A, B).

#### Clinical and biological features

**Fig. 4:**
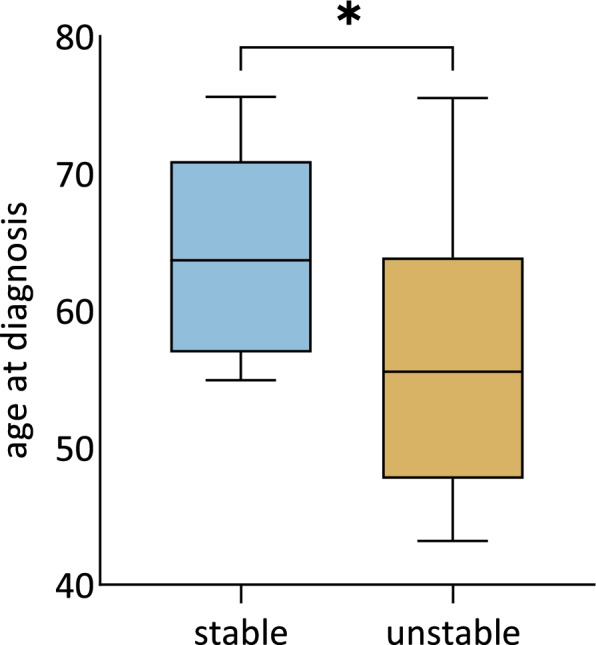
Association of relative telomere length change with the age of diagnosis (unstable *n* = 16; stable *n* = 10). Patients with unstable (i.e. shortening or prolonging) telomeres were diagnosed at younger age than patients with stable telomeres (*P* = 0.032)

All patients with changing RTLs (both shortening and extending) could be considered as one group of patients with impaired telomere maintenance resulting in unstable RTLs during the disease course. Figure 4 shows that when we examined all clinical and biological features (as listed in Supplementary Table 3) of the patients with unstable RTLs (*n* = 16), we observed that they were statistically significantly younger at diagnosis compared to the patients with stable telomeres (median 55.5 vs. 63.7; *P* = 0.032).

## Discussion

Dysregulation of telomeres is common in many cancer types and represents a means for overcoming senescence and achieving uncontrolled cell growth [46]. In CLL, short telomere length has been linked to poor prognosis [23, 31–33, 35]. Here, we used a previously described qPCR method [40, 41] to study relative telomere length (RTL) in a large cohort of 198 CLL patients. The use of pure samples containing > 98% of CLL cells enabled us to follow the RTL very accurately. We confirmed that RTL in CLL patients was generally shorter compared to the control DNA pooled from healthy donors. Short telomeres were associated with mutations in *ATM* gene as we reported previously [29] and with other markers of inferior prognosis such as unmutated IGHV, complex karyotype, del(17p) and del(11q), and mutations in *TP53* gene in concordance with published results [23–28, 30, 31]. The shortest detected telomeres were in patients with mutated *ATM* gene*,* while the longest in patients with 13q deletion as a sole cytogenetic aberration. Additionally, our study provided an insight into the relationship between clonal evolution of CLL cells with *TP53* gene aberrations and their telomere length. Within our cohort of 26 evol*TP53* cases evolving *TP53* mutations, we measured RTL at several time points. We demonstrated that the RTL can evolve diversely with the clonal evolution of genomic abnormalities in CLL.

Clonal evolution is an adverse event during the course of CLL and other cancers, which gives rise to subclones of cells with novel aberrations and features supporting increased fitness. In CLL, *TP53*-aberrant subclones tend to expand in disease relapse frequently and have a crucial impact on CLL patients’ prognosis and survival as we reported previously [8, 47]. Jebaraj et al*.* [27] hypothesized that telomere shortening preceded the appearance of *TP53* and *ATM* defects, and could provide selective pressure on CLL subclones favoring those with abilities of uncontrolled cell growth [27]. In our study, patients with *TP53* mutation expanding above 10% VAF later in a disease course did show a tendency towards shorter telomeres prior to *TP53* clonal expansion when compared to telomeres of the wt*TP53* cases. Thus, it was not possible to anticipate a forthcoming *TP53* mutation expansion in individual CLL cases based on the RTL.

In our evol*TP53* cohort, we performed measurements of paired samples and showed RTL changes between baseline and follow-up samples. Mansouri et al. [34] have found a high concordance between RTLs at diagnostic and follow-up CLL samples [34] suggesting that the telomere length does not change during the disease course. Recent study of Jebaraj et al*.* [27] focused on telomere length in 41 CLL patients without clonal evolution and 24 CLL patients with clonal evolution between samplings (9/24 had clonal evolution of *TP53* mutation detected by whole-exome sequencing). They identified significant telomere shortening between samplings only within patients with clonal evolution. In contrast, in our study specifically focused on patients with expanding *TP53* mutations, we detected stable RTL only in 38.6% of cases, whereas in the majority of evol*TP53* cases RTL alterations were noted. Interestingly, 4/26 patients with mutated *ATM* gene had significantly shorter RTL at baseline (*P* = 0.009), however, *ATM* mutation in 3/26 patients was not significantly associated with shorter RTL at follow-up (*P* = 0.061; data not shown).

To the best of our knowledge, our report is the first in CLL showing prolongation of RTL over time in a considerable proportion (23%) of evol*TP53* patients. RTL prolongation was especially apparent in samples with very short telomeres detected at baseline. In these cases, increased telomere length was frequently found in the corresponding follow-up samples, suggesting selective pressure on subclones with longer telomeres with presumably better fitness. In this context, the prognosis of CLL cases with RTL prolongation remains to be evaluated. It seems plausible that these cases, while having longer telomeres suggesting a better prognosis, probably overcame a critically short telomere crisis and gained a wide range of new aberrations, which is typical of inferior prognosis. Nonetheless, longer telomeres in baseline samples were not necessarily maintained at follow-up. Our findings, thus, highlight an unexpected consequence of clonal evolution leading to both shorter or longer telomeres, associated with changing proportions of CLL subclones with defined genomic abnormalities. A larger study, specifically addressing the fate of individual clones carrying certain telomere lengths and distinct cytogenetic abnormalities, is needed to elucidate the causality between the telomere length, genomic features, and a clone fitness.

To uncover the molecular mechanisms behind the RTL changes in our cohort, we studied two possible causal processes – hTERT expression and cellular proliferation. We did not confirm any clear role of hTERT in CLL telomere maintenance in concordance with others [23, 48]. Although the telomere shortening rate was previously attributed to a rapid proliferation rate [23, 31], neither the cellular proliferation, represented by lymphocyte doubling time, could explain the changing RTLs in our samples. Telomerase activity is highly restricted in normal somatic cells and was detected in normal B-lymphocytes in germinal centers [21]. CLL cells are well known to circulate between blood and lymph nodes [49]. Possibly telomerase activity in CLL could be restricted to the time when cells are staying in the supportive microenvironment of lymph nodes. Since our CLL samples are isolated from peripheral blood, we cannot anticipate the telomerase activity of CLL cells located in lymph nodes.

Interestingly, we found that patients with unstable telomeres, both shortening or prolonging, with concurrent evolution of *TP53* mutations, have a significantly lower age at diagnosis. We have not found any association of unstable telomeres with clinical or biological features. Some more complex factors of CLL disease nature in younger patients could be involved.

Finally, for the first time, we studied the interplay between tonic BCR signaling and telomere length maintenance in CLL. CLL cells depend on BCR signaling, supporting their survival, proliferation [50], therapy resistance, and gene expression [50–52]. BCR pathway is successfully targeted by novel inhibitor agents introduced in clinical practice in the last decade [12–14]. Using a targeted selection of phosphorylated kinases, we aimed to analyze different branches of BCR signaling to uncover possible associations with RTL changes. Patients with shorter telomeres had lower basal phosphorylation of ZAP-70/SYK and ERK1/2 in the baseline samples and lower basal phosphorylation of ZAP-70/SYK, ERK1/2, and p-NF-κB in the follow-up samples, indicating weakened BCR signaling at both time points. Other phosphorylated signaling components, especially BTK/ITK and PLC-γ2, showed strong, although not significant, trends towards lower basal phosphorylation in patients with shorter telomeres. This could anticipate that CLL cells with shorter telomeres are less dependent on survival and proliferative signals delivered to the cells by tonic BCR signaling.

## Conclusions

To summarize, we have performed an assessment of the telomere length in a well-defined cohort of CLL cases. Apart from confirming known associations between genomic features and telomere shortening, we focused on the relationship between telomere length and *TP53* mutation evolution. We discovered that long telomeres can shorten, while short telomeres can, in some cases, even extend their length during the clonal evolution process. Thus, our study shows that shifts in subclone equilibriums can be reflected in telomere length changes, while telomere length per se is likely not the only factor affecting the survival capacity of a clone expanding in the evolution process.

## Supplementary Information

**Additional file 1:** **Supplementary Table 1.** Antibodies used for BCR signaling activity assessment. **Supplementary Table 2.** Results of relative telomere length (RTL) quantification in the basic CLL cohort (*n* = 198). **Supplementary Table 3.** TP53 mutation evolution cohort – overview of clinico-biological features and results in baseline and follow-up samples, PART A Abbreviations: ALZ – alemtuzumab; BR - bendamustine and rituximab; CLB – chlorambucil; CR - cyclophosphamide and rituximab; del - deletion; F – female; FCR - fludarabine, cyclophosphamide, and rituximab; FISH - fluorescence *in situ* hybridization; hyper CVAD - hyperfractionated cyclophosphamide, vincristine, doxorubicin, and dexamethasone; IGHV – immunoglobulin heavy chain variable region; M – male; M – mutated; mo – months; U – unmutated; yrs – years. **Supplementary Table 3.** TP53 mutation evolution cohort – overview of clinico-biological features and results in baseline and follow-up samples, PART B Abbreviations: LTD – lymphocyte doubling time; mo – months; mut – mutated; p – phosphorylated; RTL – relative telomere length; VAF – variant allele frequency; wt – wildtype. **Supplementary Figure 1.** RTL of the entire CLL cohort was compared to the telomere length of a control DNA sample pooled from healthy individuals, arbitrarily set to RTL = 1 (green line). Median RTL of CLL cases (black line) was 0.81, range 0.46 – 1.25. **Supplementary Figure 2.** A) Rai (*n* = 130) and B) Binet (*n* = 133) stages at diagnosis in untreated CLL samples and their associations with RTL. The significant associations were *P*_0 vs III-IV_ = 0.0018; *P*_I-II vs III-IV_ = 0.0108; *P*_A vs C_ = 0.0043. **Supplementary Figure 3.** Overall survival (OS) of A) the entire CLL cohort (n = 198) divided by RTL above (“RTL long”) and below (“RTL short”) median RTL value (median RTL = 0.84; OS _long RTL_ = 119; OS _short RTL_ = 91; OS P = 0.0004); and B) of CLL patients with mutated *TP53* status (*n* = 40) divided by RTL above (“RTL long”) and below (“RTL short”) median RTL value in this subgroup (median RTL = 0.78; OS _long RTL_ = 86; OS _short RTL_ = 55; OS *P* = 0.046). **Supplementary Figure 4.** hTERT expression in A) the baseline samples (*P*_shortening vs. stable_ = 0.66; *P*_stable vs. prolonging_ = 0.08; *P*_shortening vs. prolonging_ = 0.45) and in B) the follow-up samples (*P*_shortening vs. stable_ = 0.77; *P*_stable vs. prolonging_ = 0.52; *P*_shortening vs. prolonging_ = 0.82), (RTL shortening n = 10; stable n = 10; prolonging n = 6). C) Comparison of hTERT values in serial samples. hTERT expression did not associate with RTL prolongation. **Supplementary Figure 5.** Lymphocyte doubling time did not correlate with a change of RTL time during the disease course (Pearson r = -0.175; *P* = 0.45). **Supplementary Figure 6.** p-ZAP70/SYK significantly correlated with p-ERK1/2 in A) baseline samples (Pearson R = 0.82; P = 0.002) and B) follow-up samples (Pearson R = 0.92; *P* < 0.001).

## Data Availability

The data used during the current study are available from the corresponding author on reasonable request.

## Abbreviations

CLL: Chronic lymphocytic leukemia
evolTP53: Clonal evolution of TP53 mutations
HBB: β-Globin
hTERT: Human telomerase reverse transcriptase
LDT: Lymphocyte doubling time
MFI: Median fluorescent intensity
qPCR: Quantitative polymerase chain reaction
RTL: Relative telomere length
VAF: Variant allelic frequency

## References

[1] Puiggros A, Blanco G, Espinet B (2014). Genetic abnormalities in chronic lymphocytic leukemia: Where we are and where we go.

[2] Guièze R, Wu CJ (2015). Genomic and epigenomic heterogeneity in chronic lymphocytic leukemia. Blood Am Soc of Hematol.

[3] Baliakas P, Jeromin S, Iskas M, Puiggros A, Plevova K, Nguyen-Khac F (2019). Cytogenetic complexity in chronic lymphocytic leukemia: Definitions, associations, and clinical impact. Blood Am Soc of Hematol.

[4] Hernández-Sánchez M, Kotaskova J, Rodríguez AE, Radova L, Tamborero D, Abáigar M (2019). CLL cells cumulate genetic aberrations prior to the first therapy even in outwardly inactive disease phase. Leukemia Nature Publishing Group.

[5] Schuh A, Becq J, Humphray S, Alexa A, Burns A, Clifford R (2012). Monitoring chronic lymphocytic leukemia progression by whole genome sequencing reveals heterogeneous clonal evolution patterns. Blood.

[6] Zenz T, Eichhorst B, Busch R, Denzel T, Häbe S, Winkler D (2010). TP53 mutation and survival in chronic lymphocytic leukemia. J Clin Oncol.

[7] Rossi D, Cerri M, Deambrogi C, Sozzi E, Cresta S, Rasi S (2009). The prognostic value of TP53 mutations in chronic lymphocytic leukemia is independent of Del17p13: Implications for overall survival and chemorefractoriness. Clin Cancer Res. Am Association for Cancer Research.

[8] Malcikova J, Stano-Kozubik K, Tichy B, Kantorova B, Pavlova S, Tom N (2015). Detailed analysis of therapy-driven clonal evolution of TP53 mutations in chronic lymphocytic leukemia. Leukemia.

[9] Landau DA, Tausch E, Taylor-Weiner AN, Stewart C, Reiter JG, Bahlo J (2015). Mutations driving CLL and their evolution in progression and relapse. Nature.

[10] Hallek M, Cheson BD, Catovsky D, Caligaris-Cappio F, Dighiero G, Döhner H (2008). Guidelines for the diagnosis and treatment of chronic lymphocytic leukemia: A report from the International Workshop on Chronic Lymphocytic Leukemia updating the National Cancer Institute-Working Group 1996 guidelines. Blood Am Soc of Hematol.

[11] Stilgenbauer S, Schnaiter A, Paschka P, Zenz T, Rossi M, Döhner K (2014). Gene mutations and treatment outcome in chronic lymphocytic leukemia: Results from the CLL8 trial. Blood Am Soc of Hematol.

[12] Byrd JC, Furman RR, Coutre SE, Flinn IW, Burger JA, Blum KA (2013). Targeting BTK with Ibrutinib in Relapsed Chronic Lymphocytic Leukemia. N Engl J Med.

[13] Furman RR, Sharman JP, Coutre SE, Cheson BD, Pagel JM, Hillmen P (2014). Idelalisib and Rituximab in Relapsed Chronic Lymphocytic Leukemia. N Engl J Med Massachussetts Med Soc.

[14] Burger JA, Tedeschi A, Barr PM, Robak T, Owen C, Ghia P (2015). Ibrutinib as Initial Therapy for Patients with Chronic Lymphocytic Leukemia. N Engl J Med Massachussetts Med Soc.

[15] Roberts AW, Davids MS, Pagel JM, Kahl BS, Puvvada SD, Gerecitano JF (2016). Targeting BCL2 with Venetoclax in Relapsed Chronic Lymphocytic Leukemia. N Engl J Med Massachussetts Med Soc.

[16] Okuda K, Bardeguez A, Gardner JP, Rodriguez P, Ganesh V, Kimura M (2002). Telomere length in the newborn. Pediatr Res Lippincott Williams and Wilkins.

[17] Lin TT, Norris K, Heppel NH, Pratt G, Allan JM, Allsup DJ (2014). Telomere dysfunction accurately predicts clinical outcome in chronic lymphocytic leukaemia, even in patients with early stage disease. Br J Haematol Blackwell Publishing Ltd.

[18] Allsopp RC, Vaziri H, Patterson C, Goldstein S, Younglai EV, Futcher AB (1992). Telomere length predicts replicative capacity of human fibroblasts. Proc Natl Acad Sci U S A.

[19] Collins K, Mitchell JR (2002). Telomerase in the human organism. Oncogene.

[20] Wright WE, Shay JW (2005). Telomere biology in aging and cancer. J Am Geriatr Soc.

[21] Norrback KF, Dahlenborg K, Carlsson R, Roos G (1996). Telomerase activation in normal B lymphocytes and non-Hodgkin’s lymphomas. Blood.

[22] Meyerson M, Counter CM, Eaton EN, Ellisen LW, Steiner P, Caddle SD (1997). hEST2, the putative human telomerase catalytic subunit gene, is up- regulated in tumor cells and during immortalization. Cell.

[23] Damle RN, Batliwalla FM, Ghiotto F, Valetto A, Albesiano E, Sison C (2004). Telomere length and telomerase activity delineate distinctive replicative features of the B-CLL subgroups defined by immunoglobulin V gene mutations. Blood.

[24] Rossi D, Bodoni CL, Genuardi E, Monitillo L, Drandi D, Cerri M (2009). Telomere length is an independent predictor of survival, treatment requirement and Richter’s syndrome transformation in chronic lymphocytic leukemia. Leukemia Nature Publishing Group.

[25] Dos Santos P, Panero J, Palau Nagore V, Stanganelli C, Bezares RF, Slavutsky I (2015). Telomere shortening associated with increased genomic complexity in chronic lymphocytic leukemia. Tumor Biol.

[26] Roos G, Kröber A, Grabowski P, Kienle D, Bühler A, Döhner H (2008). Short telomeres are associated with genetic complexity, high-risk genomic aberrations, and short survival in chronic lymphocytic leukemia. Blood.

[27] Jebaraj BMC, Tausch E, Landau DA, Bahlo J, Robrecht S, Taylor-Weiner AN (2019). Short telomeres are associated with inferior outcome, genomic complexity, and clonal evolution in chronic lymphocytic leukemia. Leukemia.

[28] Britt-Compton B, Lin TT, Ahmed G, Weston V, Jones RE, Fegan C (2012). Extreme telomere erosion in ATM-mutated and 11q-deleted CLL patients is independent of disease stage. Leukemia.

[29] Navrkalova V, Young E, Baliakas P, Radova L, Sutton LA, Plevova K (2016). ATM mutations in major stereotyped subsets of chronic lymphocytic leukemia: Enrichment in subset #2 is associated with markedly short telomeres. Haematologica.

[30] Guièze R, Pages M, Véronèse L, Combes P, Lemal R, Gay-bellile M (2016). Telomere status in chronic lymphocytic leukemia with TP53 disruption. Oncotarget Impact Journals, LLC.

[31] Rampazzo E, Bonaldi L, Trentin L, Visco C, Keppel S, Giunco S (2012). Telomere length and telomerase levels delineate subgroups of B-cell chronic lymphocytic leukemia with different biological characteristics and clinical outcomes. Haematologica.

[32] Grabowski P, Hultdin M, Karlsson K, Tobin G, Åleskog A, Thunberg U (2005). Telomere length as a prognostic parameter in chronic lymphocytic leukemia with special reference to VH gene mutation status. Blood.

[33] Strefford JC, Kadalayil L, Forster J, Rose-Zerilli MJJ, Parker A, Lin TT (2015). Telomere length predicts progression and overall survival in chronic lymphocytic leukemia: Data from the UK LRF CLL4 trial. Leukemia.

[34] Mansouri L, Grabowski P, Degerman S, Svenson U, Gunnarsson R, Cahill N (2013). Short telomere length is associated with NOTCH1/SF3B1/TP53 aberrations and poor outcome in newly diagnosed chronic lymphocytic leukemia patients. Am J Hematol.

[35] Terrin L, Trentin L, Degan M, Corradini I, Bertorelle R, Carli P (2007). Telomerase expression in B-cell chronic lymphocytic leukemia predicts survival and delineates subgroups of patients with the same igVH mutation status and different outcome. Leukemia.

[36] Kotaskova J, Tichy B, Trbusek M, Francova HS, Kabathova J, Malcikova J (2010). High expression of Lymphocyte-Activation Gene 3 (LAG3) in chronic lymphocytic leukemia cells is associated with unmutated Immunoglobulin Variable Heavy Chain Region (IGHV) gene and reduced treatment-free survival. J Mol Diagnostics Association of Molecular Pathology.

[37] Fais F, Ghiotto F, Hashimoto S, Sellars B, Valetto A, Allen SL (1998). Chronic lymphocytic leukemia B cells express restricted sets of mutated and unmutated antigen receptors. J Clin Invest Rockefeller University Press.

[38] Plevova K, Francova HS, Burckova K, Brychtova Y, Doubek M, Pavlova S (2014). Multiple productive immunoglobulin heavy chain gene rearrangements in chronic lymphocytic leukemia are mostly derived from independent clones. Haematologica.

[39] Malcikova J, Tausch E, Rossi D, Sutton LA, Soussi T, Zenz T (2018). ERIC recommendations for TP53 mutation analysis in chronic lymphocytic leukemia - Update on methodological approaches and results interpretation. Leukemia.

[40] Cawthon RM (2002). Telomere measurement by quantitative PCR. Nucleic Acids Res.

[41] O’Callaghan NJ, Dhillon VS, Thomas P, Fenech M (2008). A quantitative real-time PCR method for absolute telomere length. Biotechniques.

[42] Walsh SH, Grabowski P, Berglund M, Thunberg U, Thorsélius M, Tobin G (2007). Telomere length and correlation with histopathogenesis in B-cell leukemias/lymphomas. Eur J Haematol.

[43] JASP Team. JASP (Version 0.11.1]. 2019. p. https://jasp-stats.org.

[44] Döhner H, Stilgenbauer S, Benner A, Leupolt E, Kröber A, Bullinger L (2000). Genomic Aberrations and Survival in Chronic Lymphocytic Leukemia. N Engl J Med.

[45] Shammas MA (2011). Telomeres, lifestyle, cancer, and aging. Curr Opin Clin Nutr Metab Care NIH Public Access.

[46] Hanahan D (2011). Weinberg RA.

[47] Malcikova J, Smardova J, Rocnova L, Tichy B, Kuglik P, Vranova V (2009). Monoallelic and biallelic inactivation of TP53 gene in chronic lymphocytic leukemia: Selection, impact on survival, and response to DNA damage. Blood Am Soc of Hematol.

[48] Lin TT, Letsolo BT, Jones RE, Rowson J, Pratt G, Hewamana S (2010). Telomere dysfunction and fusion during the progression of chronic lymphocytic leukemia: Evidence for a telomere crisis. Blood Am Soc of Hematol.

[49] Burger JA, Kipps TJ (2006). CXCR4: A key receptor in the crosstalk between tumor cells and their microenvironment. Blood.

[50] Pedersen IM, Reed JC (2004). Microenvironmental interactions and survival of CLL B-cells. Leuk Lymphoma.

[51] Kurtova AV, Balakrishnan K, Chen R, Ding W, Schnabl S, Quiroga MP (2009). Diverse marrow stromal cells protect CLL cells from spontaneous and drug-induced apoptosis: Development of a reliable and reproducible system to assess stromal cell adhesion-mediated drug resistance. Blood.

[52] Herishanu Y, Pérez-Galán P, Liu D, Biancotto A, Pittaluga S, Vire B (2011). The lymph node microenvironment promotes B-cell receptor signaling, NF-κB activation, and tumor proliferation in chronic lymphocytic leukemia. Blood.

