# Supplementary material for: Evolution of TP53 abnormalities during CLL disease course is associated with telomere length changes
Source: BMC Cancer. 2022 Feb 3;22:137. doi: 10.1186/s12885-022-09221-z (PMC8812042; doi:10.1186/s12885-022-09221-z)
Supplement: Supplementary file 1 — Additional file 1: Supplementary Table 1. Antibodies used for BCR signaling activity assessment. Supplementary Table 2. Results of relative telomere length (RTL) quantification in the basic CLL cohort (n = 198). Supplementary Table 3. TP53 mutation evolution cohort – overview of clinico-biological features and results in baseline and follow-up samples, PART A Abbreviations: ALZ – alemtuzumab; BR - bendamustine and rituximab; CLB – chlorambucil; CR - cyclophosphamide and rituximab; del - deletion; F – female; FCR - fludarabine, cyclophosphamide, and rituximab; FISH - fluorescence in situ hybridization; hyper CVAD - hyperfractionated cyclophosphamide, vincristine, doxorubicin, and dexamethasone; IGHV – immunoglobulin heavy chain variable region; M – male; M – mutated; mo – months; U – unmutated; yrs – years. Supplementary Table 3. TP53 mutation evolution cohort – overview of clinico-biological features and results in baseline and follow-up samples, PART B Abbreviations: LTD – lymphocyte doubling time; mo – months; mut – mutated; p – phosphorylated; RTL – relative telomere length; VAF – variant allele frequency; wt – wildtype. Supplementary Figure 1. RTL of the entire CLL cohort was compared to the telomere length of a control DNA sample pooled from healthy individuals, arbitrarily set to RTL = 1 (green line). Median RTL of CLL cases (black line) was 0.81, range 0.46 – 1.25. Supplementary Figure 2. A) Rai (n = 130) and B) Binet (n = 133) stages at diagnosis in untreated CLL samples and their associations with RTL. The significant associations were P 0 vs III-IV = 0.0018; P I-II vs III-IV = 0.0108; P A vs C = 0.0043. Supplementary Figure 3. Overall survival (OS) of A) the entire CLL cohort (n = 198) divided by RTL above (“RTL long”) and below (“RTL short”) median RTL value (median RTL = 0.84; OS long RTL = 119; OS short RTL = 91; OS P = 0.0004); and B) of CLL patients with mutated TP53 status (n = 40) divided by RTL above (“RTL long”) and below (“RTL short”) median RTL [file 12885_2022_9221_MOESM1_ESM.docx]

Supplementary material

Article title:

Evolution of TP53 abnormalities during CLL disease course is associated with telomere length changes

Names of authors:

Helena Olbertova^1, 2^, Karla Plevova^1, 2, 3^, Sarka Pavlova^1, 2^, Jitka Malcikova^1, 2^, Jana Kotaskova^1, 2^, Kamila Stranska^1, 2^, Michaela Spunarova^2^, Martin Trbusek^2^, Veronika Navrkalova^1,2^, Barbara Dvorackova^2^, Nikola Tom^1^, Karol Pal^1^, Marie Jarosova^2, 3^, Yvona Brychtova^2^, Anna Panovska^2^, Michael Doubek^1, 2, 3^, Sarka Pospisilova^1, 2, 3^

**Supplementary Table 1:** Antibodies used for BCR signaling activity assessment.

| Antibody | Manufacturer | Cat. no. |
| --- | --- | --- |
| BV421 Mouse Anti-Akt (pS473) | **BD Biosciences** | 562599 |
| Phospho-BTK/ITK (Tyr551, Tyr511) Monoclonal Antibody (M4G3LN), APC | eBioscience | 17-9015-42 |
| BV421 Mouse Anti-ERK1/2 (pT202/pY204) | **BD Biosciences** | 562981 |
| Phospho-IKKα/β (Ser176/180) (16A6) Rabbit mAb (PE Conjugate) | Cell Signaling Technology | 14938S |
| PE-Cy™7 Mouse anti-NF-κB p65 (pS529) | **BD Biosciences** | 560335 |
| Alexa Fluor® 488 Mouse Anti-p38 MAPK (pT180/pY182) | **BD Biosciences** | 612594 |
| Alexa Fluor® 647 Mouse anti-PLC-γ2 (pY759) | **BD Biosciences** | 558498 |
| PE-Cy™7 Mouse Anti-ZAP70 (pY319)/Syk (Y352) | **BD Biosciences** | 561458 |

**Supplementary Table 2:** Results of relative telomere length (RTL) quantification in the basic CLL cohort (n = 198).

| **Parameter** | **Sample count** | **RTL median (range)** | **P-value** |  |
| --- | --- | --- | --- | --- |
| IGHV somatic hypermutation status | | | | |
| mutated | 79 (39.9%) | 0.94 (0.46-1.22) | **<0.001** |  |
| unmutated | 119 (60.1%) | 0.81 (0.51-1.25) |  |  |
| 17p status | | | | |
| intact | 170 (85.9%) | 0.85 (0.46-1.25) | **0.002** |  |
| deleted | 28 (14.1%) | 0.78 (0.51-0.94) |  |  |
| 11q status | | | | |
| intact | 153 (77.3%) | 0.86 (0.46-1.25) | **<0.001** |  |
| deleted | 45 (22.7%) | 0.76 (0.52-1.13) |  |  |
| 13q status as a sole aberration | | | | |
| no | 128 (64.6%) | 0.82 (0.51-1.25) | **<0.001** |  |
| yes | 70 (35.4%) | 0.93 (0.46-1.22) |  |  |
| Complex karyotype (>=3 changes) | | | | |
| no | 122 (72.2%) | 0.85 (0.46-1.25) | **0.001** |  |
| yes | 47 (27.8%) | 0.78 (0.51-1.09) |  |  |
| *ATM* mutation > 10% VAF | | | | |
| no | 159 (81.1%) | 0.86 (0.52-1.25) | **<0.001** |  |
| yes | 37 (18.9%) | 0.69 (0.46-1.13) |  |  |
| *TP53* mutation > 10% VAF | | | |  |
| no | 158 (79.8%) | 0.86 (0.46-1.25) | **0.001** |  |
| yes | 40 (20.2%) | 0.78 (0.51-1.02) |  |  |
| Previous treatment | | | |  |
| no | 153 (77.3%) | 0.85 (0.46-1.25) | 0.962 |  |
| yes | 45 (22.7%) | 0.83 (0.52-1.21) |  |  |

**Supplementary Table 3:** *TP53* mutation evolution cohort – overview of clinico-biological features and results in baseline and follow-up samples, **PART A**

Abbreviations: ALZ – alemtuzumab; BR - bendamustine and rituximab; CLB – chlorambucil; CR - cyclophosphamide and rituximab; del - deletion; F – female; FCR - fludarabine, cyclophosphamide, and rituximab; FISH - fluorescence *in situ* hybridization; hyper CVAD - hyperfractionated cyclophosphamide, vincristine, doxorubicin, and dexamethasone; IGHV – immunoglobulin heavy chain variable region; M – male; M – mutated; mo – months; U – unmutated; yrs – years.

**Supplementary Table 3:** *TP53* mutation evolution cohort – overview of clinico-biological features and results in baseline and follow-up samples, **PART B**

Abbreviations: LTD – lymphocyte doubling time; mo – months; mut – mutated; p – phosphorylated; RTL – relative telomere length; VAF – variant allele frequency; wt – wildtype.

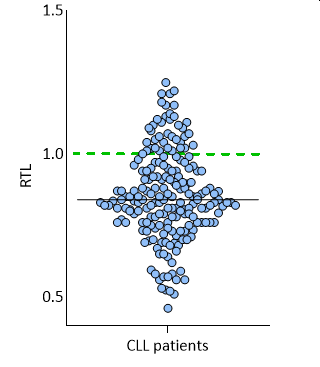
**Supplementary Figure 1: RTL of the entire CLL cohort was compared to the telomere length of a control DNA sample** pooled from healthy individuals, arbitrarily set to RTL = 1 (green line). Median RTL of CLL cases (black line) was 0.81, range 0.46 – 1.25.


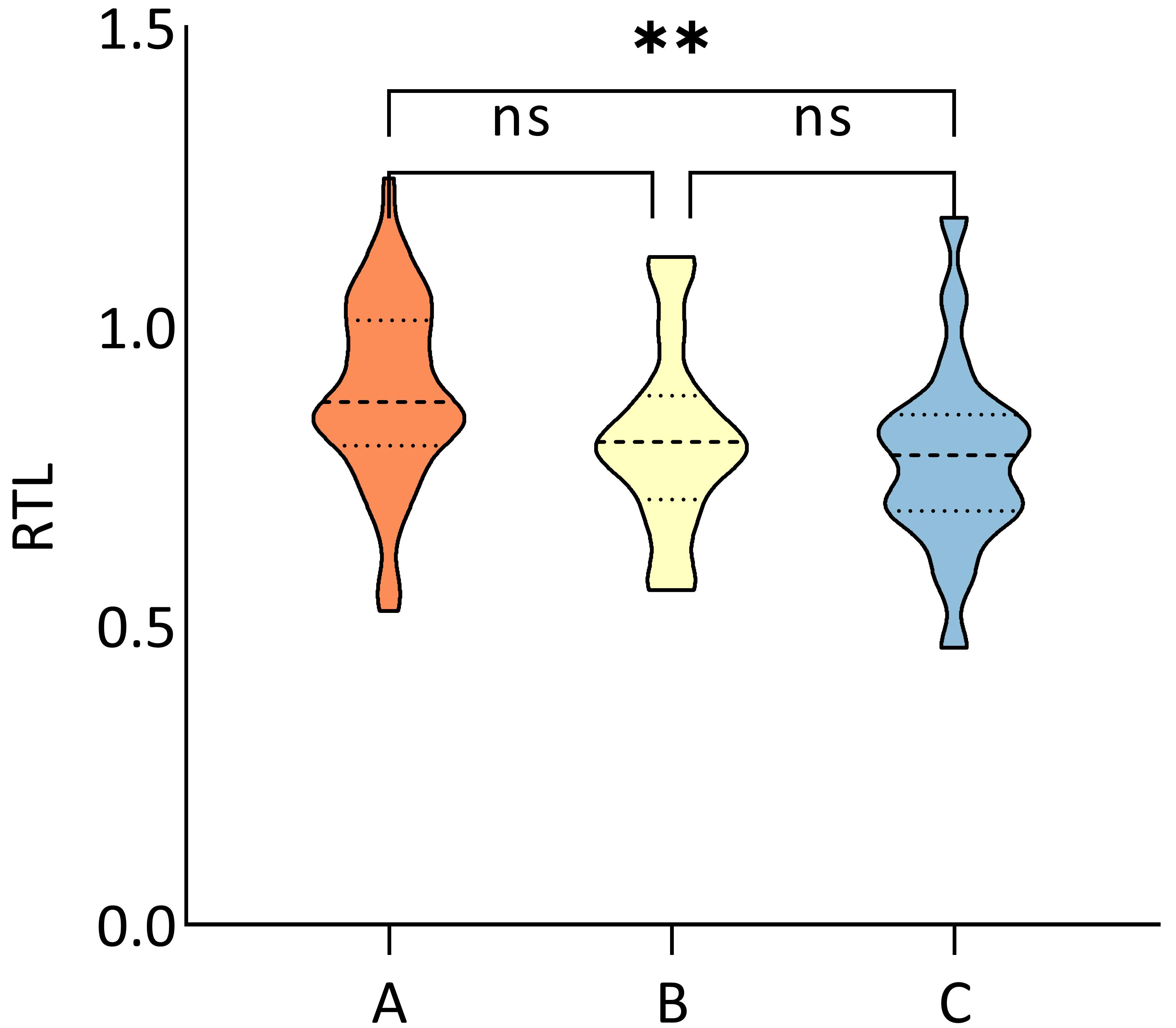

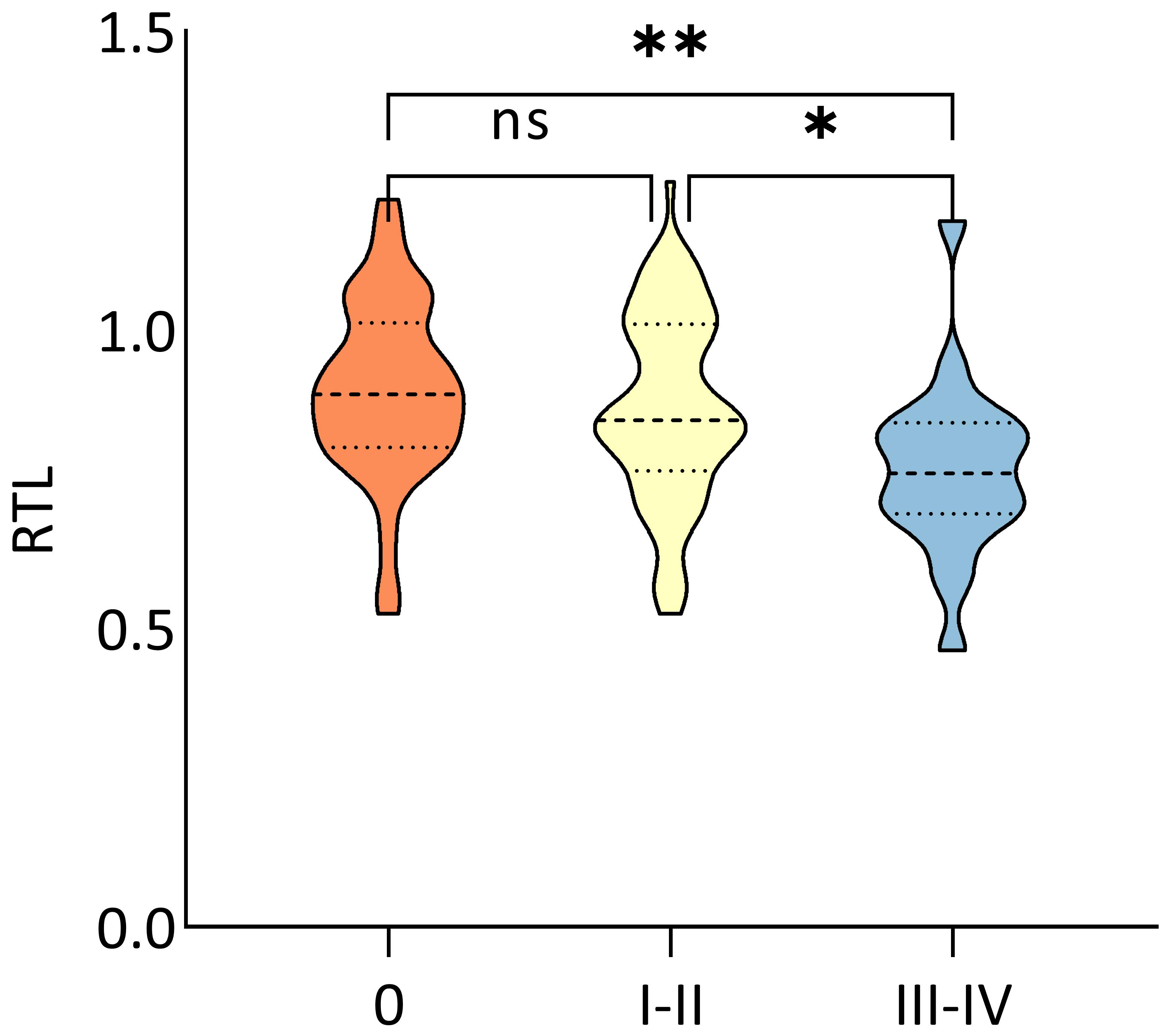
**A B**

**Supplementary Figure 2:** **A)** Rai (n = 130) and **B)** Binet (n = 133) stages at diagnosis in untreated CLL samples and their associations with RTL. The significant associations were P _0 vs III-IV_ = 0.0018; P _I-II vs III-IV_ = 0.0108; P _A vs C_ = 0.0043.

**A**   **B**


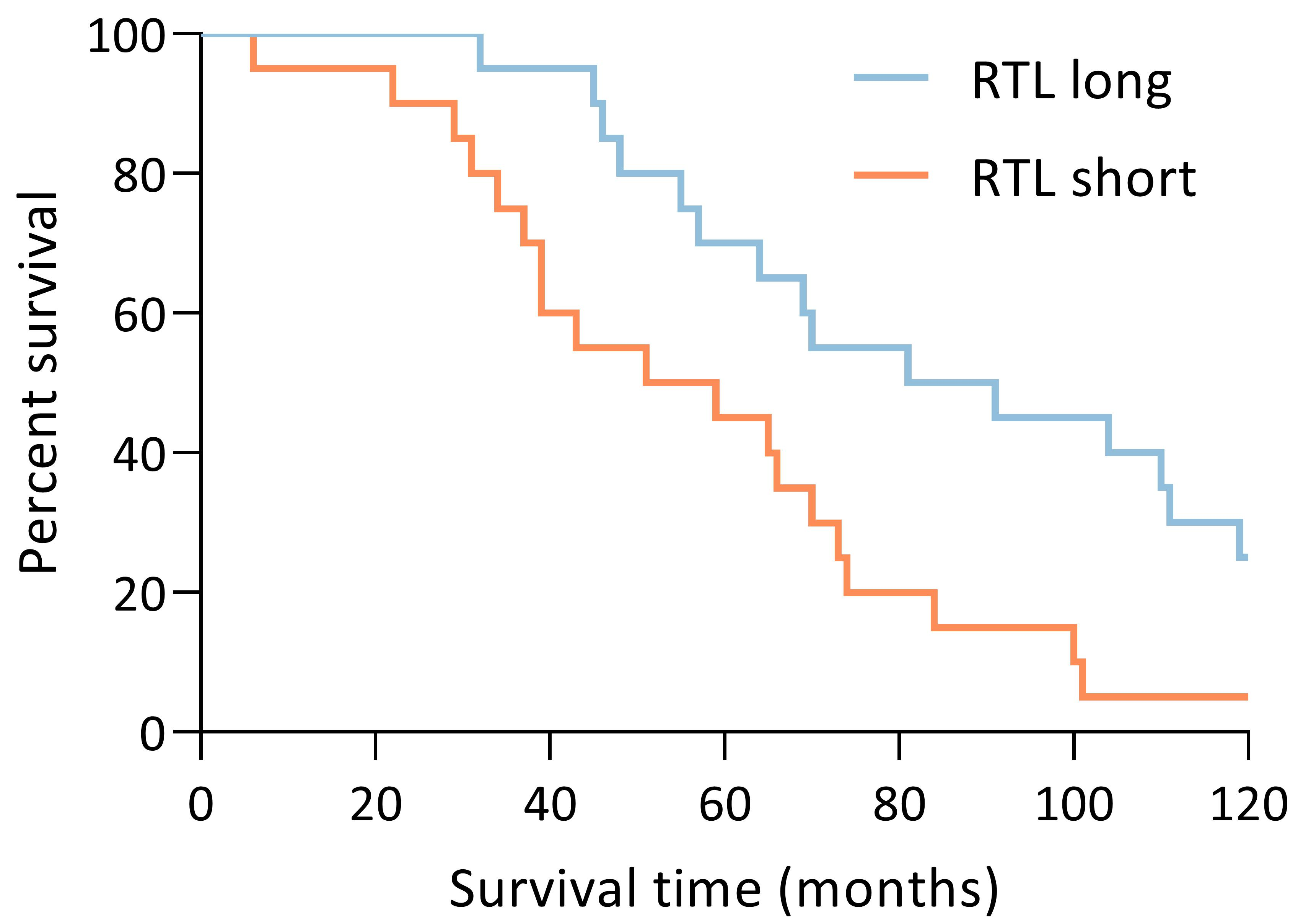


P = 0.046


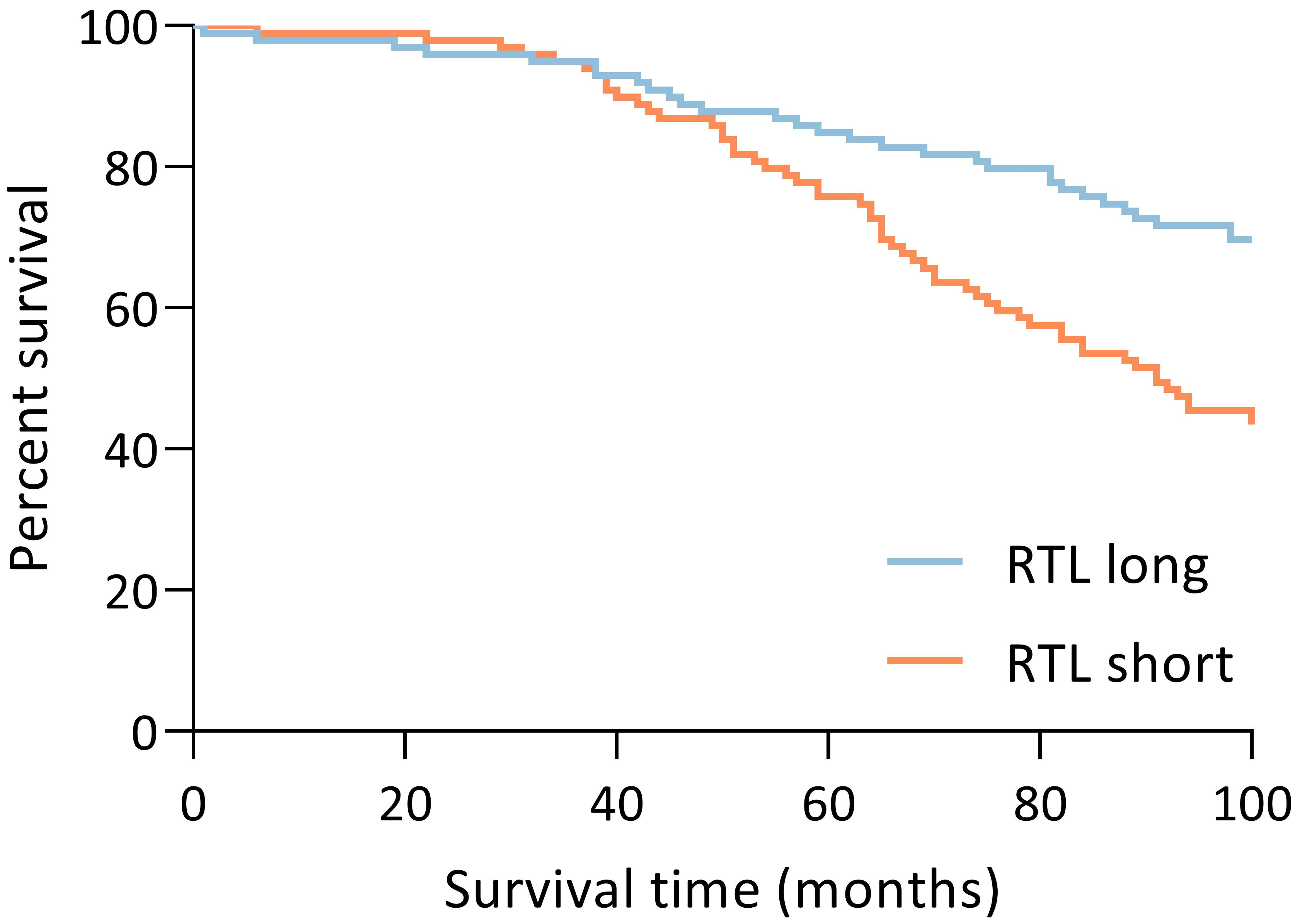


P = 0.0004

**Supplementary Figure 3:** Overall survival (OS) of **A)** the entire CLL cohort (n = 198) divided by RTL above (“RTL long”) and below (“RTL short”) median RTL value (median RTL = 0.84; OS _long RTL_ = 119; OS _short RTL_ = 91; OS P = 0.0004); and **B)** of CLL patients with mutated *TP53* status (n = 40) divided by RTL above (“RTL long”) and below (“RTL short”) median RTL value in this subgroup (median RTL = 0.78; OS _long RTL_ = 86; OS _short RTL_ = 55; OS P = 0.046).

**
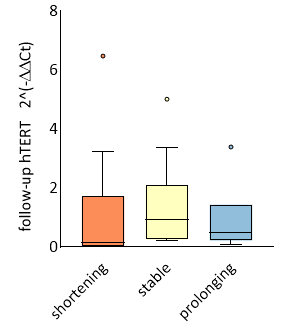

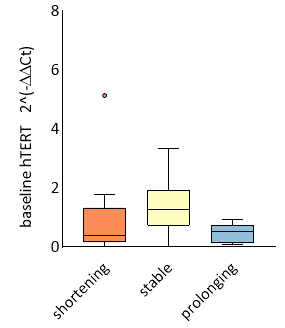
A B**

**C**


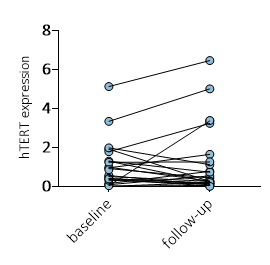


**Supplementary Figure 4: hTERT expression** in **A)** the baseline samples (P _shortening vs. stable_ = 0.66; P _stable vs. prolonging_ = 0.08; P _shortening vs. prolonging_ = 0.45) and in **B)** the follow-up samples (P _shortening vs. stable_ = 0.77; P _stable vs. prolonging_ = 0.52; P _shortening vs. prolonging_ = 0.82), (RTL shortening n = 10; stable n = 10; prolonging n = 6).
**C)** Comparison of hTERT values in serial samples. hTERT expression did not associate with RTL prolongation.

**Supplementary Figure 5: Lymphocyte doubling time** did not correlate with a change of RTL time during the disease course (Pearson r = -0.175; P = 0.45).

**A B**

**Supplementary Figure 6: p-ZAP70/SYK significantly correlated with p-ERK1/2** in **A)** baseline samples (Pearson R = 0.82; P = 0.002) and **B)** follow-up samples (Pearson R = 0.92; P < 0.001).
